# Beyond Patient Reported Pain: Perfusion Magnetic Resonance Imaging Demonstrates Reproducible Cerebral Representation of Ongoing Post-Surgical Pain

**DOI:** 10.1371/journal.pone.0017096

**Published:** 2011-02-23

**Authors:** Matthew A. Howard, Kristina Krause, Nadine Khawaja, Nathalie Massat, Fernando Zelaya, Gunter Schumann, John P. Huggins, William Vennart, Steven C. R. Williams, Tara F. Renton

**Affiliations:** 1 Department of Neuroimaging, Institute of Psychiatry, Kings College London, London, United Kingdom; 2 MRC Social, Genetic and Developmental Psychiatry Centre, Institute of Psychiatry, Kings College London, London, United Kingdom; 3 Kings College London Dental Institute, London, United Kingdom; 4 Global Research and Development, Pfizer Limited, Sandwich, Kent, United Kingdom; James Cook University, Australia

## Abstract

Development of treatments for acute and chronic pain conditions remains a challenge, with an unmet need for improved sensitivity and reproducibility in measuring pain in patients. Here we used pulsed-continuous arterial spin-labelling [pCASL], a relatively novel perfusion magnetic-resonance imaging technique, in conjunction with a commonly-used post-surgical model, to measure changes in regional cerebral blood flow [rCBF] associated with the experience of being in ongoing pain. We demonstrate repeatable, reproducible assessment of ongoing pain that is independent of patient self-report. In a cross-over trial design, 16 participants requiring bilateral removal of lower-jaw third molars underwent pain-free pre-surgical pCASL scans. Following extraction of either left or right tooth, repeat scans were acquired during post-operative ongoing pain. When pain-free following surgical recovery, the pre/post-surgical scanning procedure was repeated for the remaining tooth. Voxelwise statistical comparison of pre and post-surgical scans was performed to reveal rCBF changes representing ongoing pain. In addition, rCBF values in predefined pain and control brain regions were obtained. rCBF increases (5–10%) representing post-surgical ongoing pain were identified bilaterally in a network including primary and secondary somatosensory, insula and cingulate cortices, thalamus, amygdala, hippocampus, midbrain and brainstem (including trigeminal ganglion and principal-sensory nucleus), but not in a control region in visual cortex. rCBF changes were reproducible, with no rCBF differences identified across scans within-session or between post-surgical pain sessions. This is the first report of the cerebral representation of ongoing post-surgical pain without the need for exogenous tracers. Regions of rCBF increases are plausibly associated with pain and the technique is reproducible, providing an attractive proposition for testing interventions for on-going pain that do not rely solely on patient self-report. Our findings have the potential to improve our understanding of the cerebral representation of persistent painful conditions, leading to improved identification of specific patient sub-types and implementation of mechanism-based treatments.

## Introduction

As many as 80% of individuals experience moderate to severe post-operative pain[1] and intractable pain in patients with cancer, diabetes and HIV is a major healthcare concern[2]. The breadth of available treatments for pain control remains limited with an over-reliance on opiate-based medication[3]. Without a recordable biological marker for pain, decades of analgesic trials have relied largely on patients' own reports to describe location, intensity and quality of their pain. Standardised psychometric techniques have been developed, but inter-individual variability in pain reporting has often been incorrectly viewed as artefactual[4], rather than representing true differences in pain experience. According to a bio-psychosocial interpretation of pain[5], individual differences in pain response are likely to include effects of concurrent pathophysiology, cognitive and affective strategies and confounding effects of co-medications[6]. Compounded by a failure to report null findings, the search for novel analgesics remains slow and expensive. It has been suggested that performance issues inherent in traditional analgesic development have been stymied by continuing to use the “evaluation tools and infrastructure of the last century to develop this century's drug therapy”[3]. With this in mind, novel indices for measuring pain are required; ideally they should relate to an underlying aspect of pain transduction, take account of bio-psycho-social factors and translate between human and preclinical studies[6].

Modern neuroimaging techniques, such as Positron Emission Tomography (PET) and functional Magnetic Resonance Imaging (fMRI), show great promise in the development of novel measurement techniques, allowing non-invasive investigation of the cerebral mechanisms underpinning the pain experience. Many imaging studies to date, however, have relied on ‘experimental pain’ models using healthy volunteers to derive brain responses to acute, repeated, short-duration nociceptive stimuli (reviewed in[7], [8]). For ethical reasons, human experimental pain paradigms are often expressly designed to provide a highly controllable, psychophysically constrained stimulus that minimises tissue damage. As a result, brain responses to such stimuli are highly unlikely to account for the physiological changes that result from tissue trauma[9]. In addition, neurological sequelae that relate uniquely to individual chronic pain conditions[10], [11], [12] are largely impossible to represent in experimental models of pain in healthy controls; a fact reflected in the increasing reports of neuroimaging investigations in patients with persistent pain[13]. Both post-traumatic pain and chronic painful conditions are perceived as having an ongoing painful component. By contrast, the majority of pain-imaging studies have relied on the statistical comparison of a repeated nociceptive event with interspersed ‘rest’ or ‘control’ states derived within the same experimental session. As a result, many of these studies to date have been ill-suited to investigation of ongoing pain that cannot be modulated under experimental control within-session[14].

Compared to studies examining responses to evoked pain, there are relatively few neuroimaging reports describing the cerebral representation of ongoing pain; fewer still describe clinical ongoing pain. There are several reports using PET, for example [15], [16], [17], [18], [19] but rather than examining the ongoing clinical pain *per se*, several of these studies have examined CBF changes in response to an experimentally-derived nociceptive stimulus in addition to any ongoing background pain. Further, safety considerations, availability, expense, small group sizes and inferior temporal and spatial resolution (compared to fMRI), have limited the impact of their findings. Similarly, reports using Blood Oxygenation Level Dependent [BOLD] fMRI, for example[20], [21], have examined the relationship between changes in participants' self-reported pain and BOLD signal intensity, rather than examination of the BOLD signal alone, producing results confounded by motor responses underpinning participants' continuous online pain ratings. Others have used BOLD fMRI to examine inter-relationships in resting-state BOLD signal time series information between brain regions, known as functional connectivity analysis[22], [23]. Perhaps most importantly, conventional BOLD-fMRI paradigms are most sensitive to signal changes over several seconds and are less suitable for examining pain responses lasting many minutes[24] or for monitoring long-term treatment effects[25]. By contrast, perfusion MRI methodologies such as arterial spin labelling (ASL)[26], [27] may be preferable for the study of behaviours or states over the course of minutes as opposed to seconds. ASL has already been documented as an ideal methodology for the central investigation of ongoing, non-paroxysmal pain[14]. The methodology provides quantitative, reproducible rCBF measurements throughout the brain and has superior noise-power characteristics, compared to fMRI, in within-subject designs with a task periodicity of 120 seconds or greater[28]. The application of ASL to the study of pain remains in its infancy[29], [30]; to the best of our knowledge there has yet to be a report of the application of ASL to ongoing, clinically-relevant pain.

Here we assess the validity of pulsed-continuous ASL [pCASL] [31] as a quantitative, reproducible marker of ongoing post-surgical pain. We applied the most commonly employed clinical pain model used in trials of analgesics such as non-steroidal anti-inflammatory drugs and opiates, the third molar extraction (TME) model[32], [33]. In the TME model, healthy participants, with no prior history of chronic painful disease other than recurrent, intermittent pericoronitis of their third molars, are recruited. As a result, participants are unaffected by confounding variables such as heterogeneity in pain distribution, concomitant medication and pathology and participants can be initially assessed while asymptomatic and completely pain-free. Often bilateral, similarly-positioned wisdom teeth require extraction that are matched for surgical difficulty, resulting in reproducible amounts of moderate-to-severe post-surgical pain following each unilateral extraction[34]. Reproducibility of pain response renders the model ideal for ‘cross-over’ placebo-controlled analgesic trials. In addition a recent meta-analysis reported that TME-derived assessments of analgesic efficacy could be extrapolated to other forms of post-surgical pain[32], demonstrating the broad utility of the model.

In this study we applied pCASL to the challenge of representing the cerebral basis of ongoing pain. We imposed three constraints, namely that the ongoing pain experience was induced by genuine tissue damage, could not be modulated by the experimenter within a single session, and that assessments of ongoing pain could be repeated to fulfil the requirements of a cross-over trial design. We demonstrate quantitative, reproducible rCBF increases that represent the experience of being in ongoing pain following TME including those in a network of brain regions specified *a priori*. Further, we provide novel insights into the central representation of post-surgical trigeminal pain in humans. Our findings are discussed in terms of their potential impact on development of novel interventions for treatment of acute and chronic pain conditions and how the pCASL technique might be utilised in translational research.

## Methods

### Ethics Statement

This study was approved by Kings College Hospital Research Ethics Committee (REC Reference 07/H0808/115).

### Subjects and Materials

16 right-handed, healthy male volunteers aged 20–41, (mean = 26.4 years) provided informed consent to participate in the study. Females were excluded due to potential variability in the phase of the menstrual cycle affecting reproducibility of the response to post-surgical pain[35]. All participants presented with bilateral recurrent pericoronitis and fulfilled NICE guidelines for extraction of lower-jaw left and right third molars[36].

### Experimental Design

Participants were scanned on five separate occasions (S1–S5); screening/familiarisation (S1), pre-surgical (S2) and post-surgical sessions (S3) for the first extraction and pre-surgical (S4) and post-surgical (S5) sessions for the second extraction. An interval of at least two weeks separated S3 and S4, following complete recovery from the first surgery. Order of left and right tooth extraction was balanced and pseudo-randomised across the group. At each session, pulse rate and blood pressure were recorded, an alcohol and drug-screen performed and a psychometric assessment completed. Analgesic medication (1000 mg paracetamol & 400 mg ibuprofen) was provided to participants immediately following scanning during S3 & S5.

### Procedure

At S1, standardised screening questionnaires were administered to assess presence of any pain and baseline psychometric information (see Baseline Psychometry). A short MR examination was performed for familiarisation with the imaging environment and participants received training on using a computerised, joystick-operated visual analogue scale (VAS). MR examinations during sessions S2–S6 were identical, each comprised of six separate consecutive pCASL scans, each lasting six minutes. Participants were instructed to lie still with their eyes open. Prior to and following acquisition of each rCBF map, participants subjectively rated pain intensity and alertness using a computerised VAS.

### Baseline Psychometry

Baseline psychometric screening assessments were performed for all participants prior to scanning at S1. Screening for depression was performed using the Center for Epidemiological Studies Depression Scale [CES-D][37], and trait and state anxiety using the State-Trait Anxiety Questionnaire [STAQ][38]. Changes in state anxiety relating to surgery were assessed at the beginning of each session. Screening for general mental health status was assessed using the Revised Symptom Checklist 90 [SCL-90-R][39], and for alcohol and drug abuse using sections 11 and 12 of the Schedules for Clinical Assessment in Neuropsychiatry [SCAN][40]. Finally, the Cognitive Coping Strategies Inventory [CCSI][41] was administered in order to assess participant coping strategies for pain. Participants with psychometric data outside published normative limits for each test were not included in the study.

### Surgery

Unilateral TME was performed under local anaesthesia (4.4 ml Lignospan Special, Septodont) using a standardized technique. Surgical difficulty was rated on a 1-5 scale[42]. Following surgery, participants were supervised for up to six hours before their post-surgical scan, during which time ratings of pain intensity were recorded using a pen-and-paper 100 mm VAS. Scanning commenced when three consecutive VAS scores greater than 30/100 mm were provided within a 30-minute period.

### Imaging Procedure

Imaging was performed on a 3 Tesla Signa HDx whole-body MR imaging system (General Electric, USA) fitted with an 8-channel, phased-array receive-only head coil. High-resolution T1- and T2-weighted MR structural sequences were acquired for radiological assessment and image registration. Resting-state rCBF measurements were made using pCASL[31], using an irradiation time of 1.5 s and post-labelling delay of 1.5 s. pCASL images were acquired using a single-shot, Fast Spin Echo readout resulting in whole-brain blood flow maps, with a spatial resolution of 1×1×3 mm.

### Image Preprocessing

Preprocessing and analysis were performed using FSL v4.1.0 [http://www.fmrib.ox.ac.uk/fsl][43]. Preprocessing prior to voxelwise analysis using a General Linear Model (GLM), consisted of skull stripping [BET], registration to the Montreal Neurological Institute (MNI) template [FLIRT] and a non-linear noise-reduction algorithm [SUSAN] to improve signal-to-noise ratio and condition the data for statistical analysis.

### Surgical and Behavioural data analysis

All surgical and behavioural data analyses were computed using GenStat v11.1 (http://www.vsni.co.uk/). Variability in perceived surgical difficulty and surgery-to-scan time between left and right tooth extractions were assessed using student's t-tests. VAS estimates of pain and alertness were fitted to a mixed effect model, with Participant and Participant-by-Session as random effects, and Session-pair (Pair 1[S2,S3]/Pair 2[S4,S5]), Surgery (Pre-surgery/Left/Right tooth post-surgery),Timepoint, and Surgery by Timepoint as fixed effects. A first-order auto-regressive (AR(1)) covariance structure was specified for the repeated measures Timepoint. Significance thresholds for all behavioural analyses were at the p<0.05 level.

### Whole brain voxel-wise analysis

Statistical analysis of pCASL data was applied at two levels using a voxelwise optimised GLM [FLAMEO]. First-level analyses were computed for each subject to create grey-matter only mean and variance images of the six individual pCASL scans acquired at each of sessions S2–S6. These images were used in a higher-level mixed effects analysis with Participant, Surgery (Presurgery/Left/Right tooth surgery) and Session-pair (Pair 1[S2,S3]/Pair 2[S4,S5]) as model terms, to assess changes in rCBF relating to post-surgical pain and rCBF differences following left, compared to right TME. Z-statistic images were thresholded using clusters determined by Z>2.3 and a corrected cluster-significance threshold of p = 0.05 according to random field theory[44].

### ROI Creation

Anatomical ROIs in MNI-template space were derived from Harvard-Oxford Cortical/Subcortical and Juelich-Histological Atlases. Based on *a priori* information regarding brain activation related to pain, ROIs were created for anterior cingulate cortex [ACC], primary [S1], and secondary [SII] somatosensory cortices, insula [INS], thalamus [THAL], amygdala [AMY] and hippocampus [HIP] in each cerebral hemisphere. Finally, an ROI was created for V5/MT, an *a priori*-defined, comparably-sized control ROI involved in visual motion perception and eye movements[45]. We hypothesised that rCBF in V5/MT would not be modulated by post-surgical pain.

### ROI Data Extraction

Two ROI datasets were created. In both datasets, the mean of the 20% voxels with greatest CBF values was computed[46] as a summary measure. In set one, ROIs were extracted from each individual CBF map acquired at for each participant at each session; these data were used to examine temporal variation in rCBF response to post-surgical pain. In set two, ROIs for each hemisphere at each session were extracted from mean images created following first level voxelwise analyses.

### ROI Analysis

All ROI analyses were performed using GenStat v11.1.

*Temporal variation within-session* rCBF values extracted from set one were plotted to examine temporal variation in rCBF value within a single session. For each ROI in each hemisphere, rCBF estimates from each pCASL scan were fitted to a mixed effect model, with Participant, Participant-by-Session, Participant-by-Session-by-Time and Participant-by-Session-by-Hemisphere as random effects, Session-Pair (Pair 1/Pair 2) as fixed effect, and a 3-way factorial of Surgery (Pre-surgery/Left/Right tooth Post-surgery) Hemiphere (Left/Right) and Timepoint (1–6). P-values were Bonferroni corrected for multiple comparisons.

### Pre/Post-surgical differences

For each ROI in each hemisphere, rCBF values for each subject in each session were fitted to a mixed effect model. Participant and Participant-by-Session were fitted as random effects, and Surgery (Pre-surgery/Left/Right-tooth post-surgery), Session-pair (Pair 1[S2,S3]/Pair 2[S4,S5]) and Hemisphere (Left/Right) were fitted as fixed effects. Significance thresholds were imposed after Bonferroni correction.

### Correlation Analysis

For each ROI, an ANCOVA model was fitted to rCBF values obtained from each hemisphere in set two. Subject was fitted as a fixed effect and VAS estimate of pain [VAS] fitted as a covariate. The model was used to calculate intra-subject correlation co-efficients (ρ_w_) for each ROI[47]. Due to the exploratory nature of these correlation analyses, multiple comparison correction was not employed.

## Results

### Surgical Outcome

There were no differences relating to site of surgery (left versus right). Perceived surgical difficulty and time taken from local anaesthesia to first CBF map did not differ between left and right surgeries (Difficulty: Left = 3.29, Right = 3.47; paired-t, p = 0.44; Time taken: Left = 210 minutes, Right = 204 minutes; paired-t, p = 0.738).

### Psychometric Outcomes

**Figure 1:**
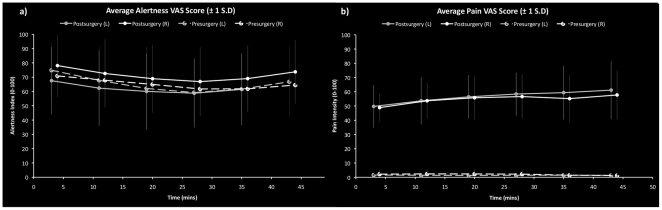
Within-scanner time courses of VAS indices of (a) perceived alertness and (b) pain experienced pre/post each pCASL scan. Each visit is plotted separately (Left tooth  =  Grey, Right Tooth  =  White; Filled circles =  Post-surgical visit, Unfilled circles  =  Pre-surgical visit; Error bars indicate ±1 Standard Deviation.

Mean alertness ratings did not differ between pre- and post-surgical MRIs (Pre-surgery = 62.36, Post-surgery = 66.4; p = 0.35), (Figure 1a). There was no session order effect (p = 0.592). Mean post-surgical pain ratings were increased compared to pre-surgical visits (Figure 1b) (Pre-surgery = 1.8, Post-surgery = 56.5; F_[1,39.6]_ = 432.99, p<0.001), but there were no differences following extraction of left, compared to right, third molars (p = 0.97). There was no session order effect (p = 0.55).

### Neuroimaging

**Figure 2:**
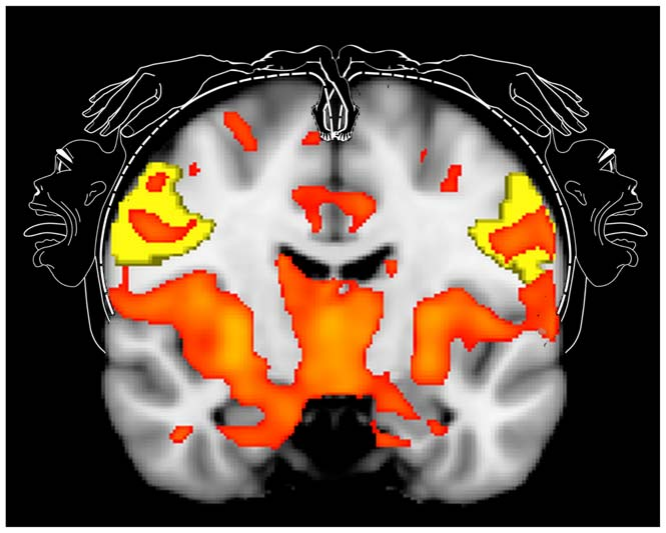
Post-surgical CBF changes in S1 relate to the classical somatotopic representation of the jaw (adapted from [**48**]). CBF increases coded in red illustrates mask image of clusters significant at the p<0.05 (corrected) level. Yellow mask illustrates S1 ROI in left and right cerebral hemispheres.

**Figure 3:**
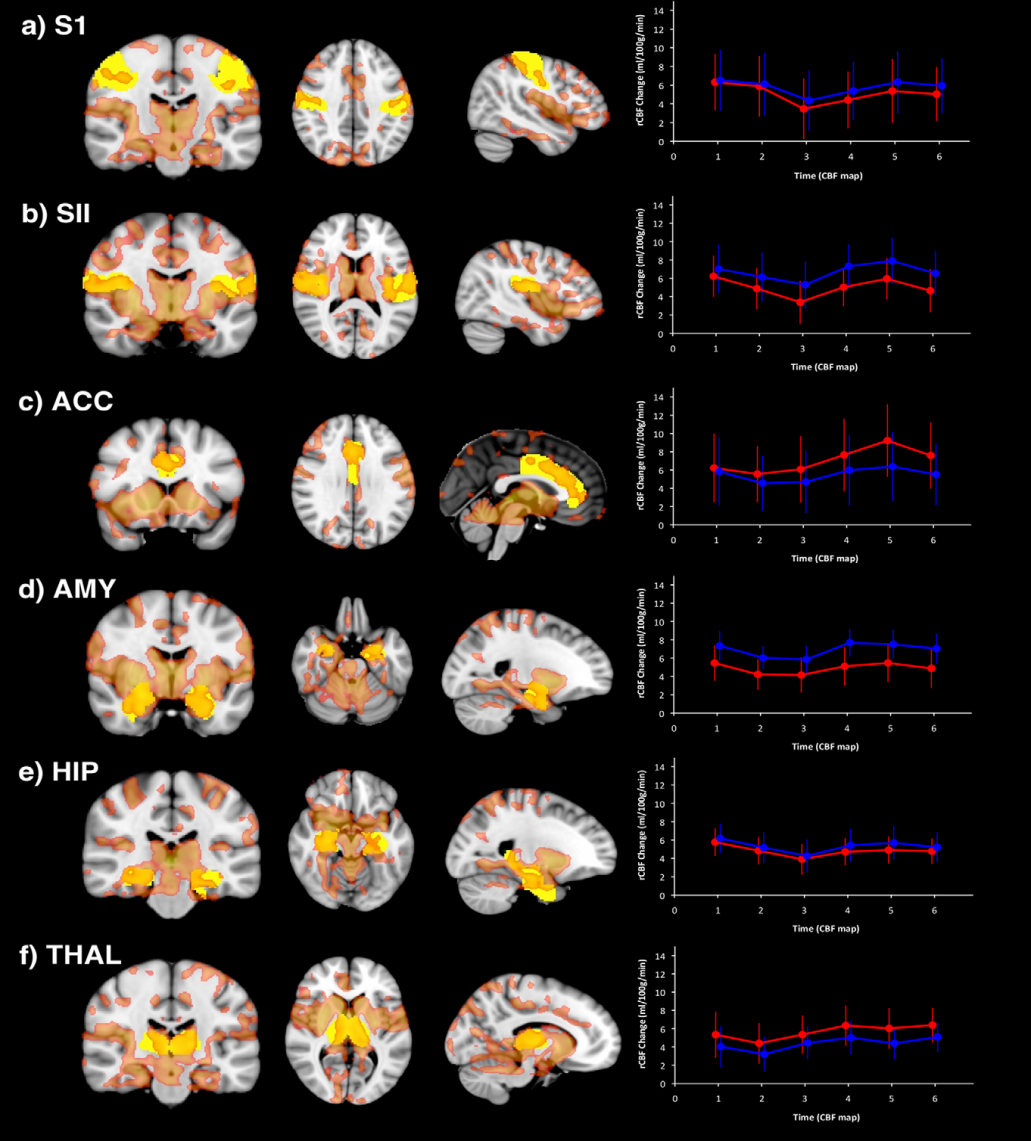
(a–f) Time courses of post-surgical rCBF increases relating to pain in each a priori-defined ROI. Cluster-corrected (p<0.05) Z-statistic map (red) indicates regional post-surgical increases in CBF relating to pain. In each row, *a priori* ROI masks are outlined in yellow. Plots at far right of each row indicate time courses of post-surgical increases in CBF (ml/100 g/min) for each ROI extracted from each individual pCASL scan (Red  =  left hemisphere, Blue  =  right hemisphere; Error bars represent ±1 Standard Error).

A distributed network of brain regions demonstrated significant increases in rCBF relating to pain following extraction of left and right third molars, compared to pain-free pre-surgical periods in the same subjects. Table 1 details each cluster in brain regions we hypothesised *a priori* would demonstrate CBF changes during post-surgical pain; for brevity, only clusters with highest Z-scores per anatomical region have been reported. We did not observe any post-surgical decreases in CBF in these regions or elsewhere. In particular, bilateral increases in rCBF during post-surgical pain were identified in postcentral gyrus, specifically the somatotopic region of S1 relating to the face/jaw[48], [49] (Figure 2; 3a), in SII (Figure 3b), extending ventrally towards posterior insula cortex and in mid/anterior insula cortices, extending towards the frontal operculum. At midline, clusters were observed bilaterally in mid-anterior cingulate cortices, (Figure 3c) extending towards perigenual cingulate cortex, and in posterior cingulate gyrus close to the splenium of the corpus callosum. In the temporal lobe, clusters were identified in amygdala (Figure 3d), extending dorsally through hippocampal/parahippocampal cortices (Figure 3e). In the thalamus, a single, bilateral interconnected cluster was identified which included pulvinar, ventral posterior, ventromedial and anterior regions at midline, extending inferiorly to include the hypothalamus (Figure 3f).

**Table 1:** Regions of increased post-surgical CBF specified *a priori* to underpin cerebral processing of pain.

Structure	Left Hemisphere				Right Hemisphere			
	Z-stat	x	y	z	Z-stat	x	y	z
Primary Somatosensory Cortex	3.41	−62	−16	42	3.17	42	−16	42
Secondary Somatosensory Cortex	3.29	−56	−14	14	3.36	54	−14	16
Thalamus	3.46	−20	−28	16	3.76	16	−36	6
Posterior Cingulate	3.02	−8	−64	12	3.38	24	−70	6
Cingulate Gyrus	3.03	−4	−32	24	3.25	10	−42	26
Mid-anterior Cingulate Gyrus	3.26	−12	6	36	3.38	14	−12	44
Anterior Cingulate	-	-	-	-	3.13	6	36	6
Hippocampus/Parahippocampus	4.05	−28	−50	−6	4	26	−44	−4
Amygdala	3.8	−30	2	−26	4.32	24	0	−14
Insula	3.47	−44	−10	6	4.1	40	−12	14

**Figure 4:**
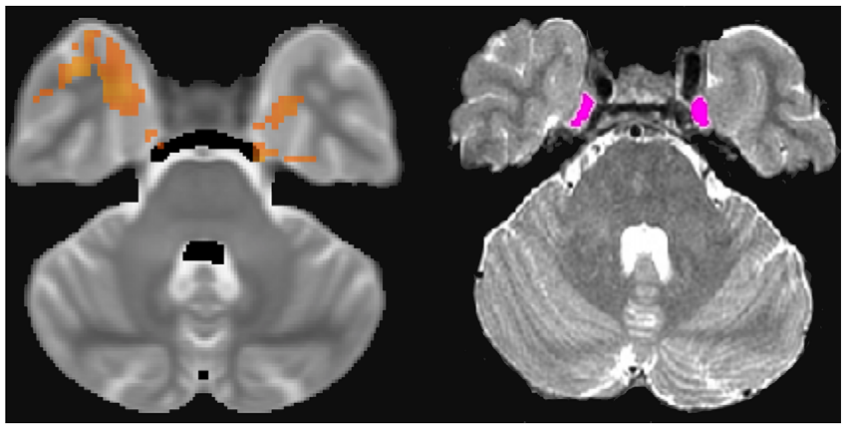
Anatomical and Functional Localisation of the Trigeminal Ganglion. (left) High resolution axial T2-weighted image illustrates Meckel's cave (magenta), the anatomical location of the trigeminal ganglion. (right) Post-surgical rCBF increases in trigeminal ganglion.

Further regions of increased post-surgical rCBF (Table 2) were identified in addition to those specified *a priori*. In the frontal lobe, clusters were identified in superior, middle, medial and orbital-frontal cortices, in precentral gyrus and superior and inferior parietal lobules bilaterally. In the temporal lobe, bilateral regions of increased CBF were identified in superior, middle inferior temporal and fusiform gyri, and in the lingual gyrus and precuneus in the occipital lobe. In the basal ganglia, clusters were identified in caudate and lentiform nuclei bilaterally. In the brainstem, increased post-surgical CBF was identified bilaterally adjacent to the lateral mid-pons, approximating to the trigeminal ganglion/roots (Figure 4), with further continuous regions of increased rCBF in mid-pons identified as principal sensory trigeminal nucleus (Vp), extending posteriorly towards bilateral anterior cerebellar hemispheres and vermis. Superior to Vp, a single cluster was observed encompassing the pontine reticular formation, ascending superiorly into midbrain reticular formation including much of the tegmentum including substantia nigra, ventral tegmental area and red nucleus, and tectum including quadrigeminal body and periaqueductal grey.

**Table 2:** Additional regions of increased post-surgical CBF not specified *a priori* to underpin central processing of pain.

Structure	Left Hemisphere				Right Hemisphere			
	Z-stat	x	y	z	Z-stat	x	y	z
Medial Frontal Gyrus	2.66	−12	38	24	2.71	8	−8	60
Superior Frontal Gyrus	2.74	−20	−4	68	2.91	34	56	28
Middle Frontal Gyrus	3.12	−34	2	66	2.78	24	−2	46
Inferior Frontal Gyrus	3.77	−36	8	−16	3.2	42	32	−12
Orbital Gyrus	-	-	-	-	2.95	4	42	−22
Rectal Gyrus	-	-	-	-	2.95	12	42	−18
Rectal Gyrus	-	-	-	-	2.86	6	32	−24
Precentral Gyrus	3.92	−60	10	0	3.49	64	8	10
Postcentral Gyrus	2.7	−28	−48	72	3.22	22	−34	66
Paracentral Lobule	3.46	10	−32	62	3.15	6	−42	72
Superior Parietal Lobule	2.92	−22	−60	56	3.2	26	−66	56
Inferior Parietal Lobule	2.64	−2	−94	26	3.35	24	−62	30
Superior Temporal Gyrus	3.78	−64	−6	4	3.77	36	8	−20
Middle Temporal Gyrus	-	-	-	-	3.25	64	−40	−10
Inferior Temporal Gyrus	-	-	-	-	3.49	38	−6	−28
Fusiform Gyrus	3.32	−36	−34	−22	3.61	36	−40	−18
Supramarginal Gyrus	-	-	-	-	2.84	−28	−46	38
Superior Occipital Gyrus	-	-	-	-	2.74	34	−88	22
Precuneus	3.22	−18	−62	30	3.42	22	−86	42
Lingual Gyrus	2.68	−20	−78	−4	3.69	18	−84	−6
Cuneus	3.17	−14	−74	16	3.01	0	−100	4
Lentiform Nucleus	4.55	−26	2	−4	4.24	30	−12	2
Caudate	3.78	−10	20	6	4.71	6	4	2
Internal Capsule	4.26	−18	20	−6	4.49	30	6	−4
Claustrum	-	-	-	-	4.31	32	0	8
Midbrain	3.22	−16	−22	−8	3.54	4	−24	−14
Trigeminal System	3.53	−18	−18	−32	-	-	-	-
Pons	2.98	−18	−28	−32	3.11	14	−30	−26
Cerebellum	3.25	−20	−46	−32	3.23	12	−56	−26
Cerebellar Lingual	-	-	-	-	3.9	2	−46	−24
Declive	-	-	-	-	3.29	50	−50	−26
Culmen	3.2	−12	−70	−12	3.68	12	−44	−24

### ROI Analysis: Temporal Variation Within Session

The anatomical location of each ROI, post-surgical CBF change and associated time courses is illustrated in Figure 3(a−f). Mixed effect model analyses in each *a priori* ROI demonstrated that no significant variation in rCBF across scans (Time) was identified within a single session. There were no other significant second or third order interactions of Time with Hemisphere, or Surgery, indicating that within-session temporal variation across pCASL scans did not differ between cerebral hemispheres, either in pre-surgical or post-surgical scanning sessions following either left or right TME. In the light of these findings, assessment of between session variation in rCBF was studied using ROIs derived from set two, the average of all 6 cASL maps acquired within a single session.

### Pre/Post surgery differences

Mixed effect models were computed for all pain and control ROIs. Main effects and interactions for ROIs are summarised in Table 3. In each pain-related ROI, rCBF increases between 5−10% were identified following TME. Following correction for multiple comparisons, significant increases in post-surgical rCBF were observed in AMY, HIP, SII, THAL, & INS ROIs, with strong trends in the same direction identified in S1 and ACC, but not in control region V5/MT. There was no effect of side of first tooth removal. A main effect of Hemisphere was observed in all ROIs, including control region V5/MT but excluding HIP, which indicated that both pre- and post-surgical rCBF values for ROIs were increased in right, compared to left hemisphere. There were no significant interactions of Hemisphere with Surgery side across all ROIs, meaning that surgery effects had the same impact on each hemisphere independently of whether left or right third molar was removed.

**Table 3:** ROI Analysis summary table.

ROI	Estimated Marginal Means			Pre-surgery vPost-surgery			Post-surgery (LvR)			Hemisphere		Session-Pair	
	Presurgery	Post-surgery [L]	Post-surgery [R]	Mean difference	F-ratio	p	Mean difference	F-ratio	p	F-ratio	p	F-ratio	p
AMY	56.6	60.2	61.3	4.2	14.45	0.000^*^	1.1	0.47	0.49	18.87	0.00	0.04	0.85
HIP	59.3	62.1	63.4	3.5	10.47	0.002*	1.3	0.73	0.40	0.57	0.45	0.08	0.78
INS	81.0	85.4	87.5	5.4	10.38	0.002*	2.1	0.79	0.38	27.23	0.00	0.00	0.99
S1	66.6	71.2	71.2	4.6	5.28	0.026	0	0.00	0.99	69.58	0.00	0.01	0.91
S2	71.9	76.2	77.2	4.8	11.02	0.002*	1.1	0.27	0.61	32.05	0.00	0.04	0.85
ACC	93.3	97.0	100.2	5.3	6.80	0.012	3.2	1.27	0.27	157.04	0.00	0.12	0.73
THAL	67.4	71.5	74.2	5.5	15.35	0.000*	2.8	1.98	0.17	18.34	0.00	0.03	0.85
V5	76.4	77.3	77.9	1.2	0.39	0.534	0.6	0.04	0.84	159.98	0.00	0.01	0.91

(Columns, left to right) ROI; Mean rCBF values from pre- and post-surgical sessions on left [L] and right [R] teeth; Mean rCBF difference between pre and post-surgical sessions; F-statistic and associated p-value for main effect of Surgery (ROIs significant after Bonferroni correction illustrated by an asterisk); comparison of rCBF differences between post-surgical sessions following left and right TME and associated F-statistic and p-value; F-statistic and associated p-value for rCBF differences between left and right cerebral hemispheres.

### Relationships Between VAS Pain Estimates and rCBF

Within-subject correlation co-efficients (ρ_w_) were computed for each ROI in each hemisphere to assess the relationship between post-surgical pain rCBF and patients' self-reported pain VAS scores. Significant linear relationships were identified in AMY, HIP, S1, SII, THAL, INS, PCC & ACC ROIs, (Table 4) but not in control region V5/MT.

**Table 4:** Within-subject correlation co-efficients (ρw) between mean rCBF and mean VAS-derived estimates of post-surgical pain in each ROI.

Structure	Left Hemisphere		Right Hemisphere	
	ρ_w_	F-prob	ρ_w_	F-prob
Amygdala	0.41	0.004	0.51	0.001
BrainStem	0.4	0.005	0.44	0.002
Hip_Form	0.42	0.003	0.46	0.001
Insula	0.35	0.014	0.48	0.001
S1	0.36	0.011	0.41	0.003
S2	0.38	0.008	0.46	0.001
ACC	0.37	0.009	0.37	0.009
Thalamus	0.47	0.001	0.46	0.001
V5	0.15	0.304	0.23	0.122

## Discussion

Using pCASL, we have demonstrated reproducible, rCBF-derived markers of ongoing, clinically-relevant pain. Increases in rCBF were established following surgery, compared to pain-free pre-surgical periods, in an un-biased voxel-wise analysis and in *a priori* hypothesised regions inherent in the central processing of pain, but not in control brain regions hypothesised to be unchanged by pain. rCBF assessments were stable within a single session and there were no between-session differences in post-surgical rCBF following extraction of left, compared to right, teeth, indicating a viable test-retest paradigm. Post-surgical CBF changes correlated with VAS estimates of self-reported pain, but only in brain regions known to underpin the processing of pain and not in a control brain region. Quantitative changes in rCBF that represent ongoing pain have potential as markers of treatment efficacy for acute and persistent painful conditions.

Our findings of rCBF increases during pain following TME provide valuable new insights into the representation of ongoing post-surgical trigeminal pain. Independently of site of removal, the pain resulting from tooth extraction is represented by a largely bilateral pattern of rCBF changes throughout the brain. No hemispheric differences in rCBF changes related to extraction were found. These findings differ from earlier pain studies using PET imaging, which have largely reported rCBF changes contralateral to the painful body-site, for example, contralateral increases in rCBF in PFC, insula cortex, and lentiform nucleus were reported following a composite third molar extraction and thermal heat pain challenge[16]. To the best of our knowledge, this is the only other neuroimaging study of pain following third molar extraction, but is difficult to relate to our findings due to the confounding effect of a nociceptive heat stimulus applied to the hand contralateral to the extracted tooth. Two recent reports using experimental pain models have highlighted the potential of ASL in pain research[29], [30]. Several findings in those studies were concordant with our own, namely, similar magnitude of CBF values in grey matter and resulting rCBF changes in response to pain in bilateral insula cortex, SII, cingulate cortex and supplementary motor area, as well as responses in S1 and thalamus. However, contrary to our own findings, responses to a tonic painful hypertonic saline stimulus produced a CBF decrease in S1, while several additional regions demonstrated a reduction in magnitude of the CBF change over the time course of the saline infusion. We speculate that such CBF decay characteristics may relate to differences not only in physiological response but also in terms of the threat value of an experimentally evoked stimulus, compared to a genuine post-surgical tissue trauma [20], [49], [50]. Differences in ASL implementation in those studies precluded further examination of CBF changes inferior to the thalamus and provided a lower spatial resolution than reported here, and further comparisons are difficult due paradigm design, body-site differences, and potentially confounding CBF changes relating to patient introspection and movements derived from providing VAS estimates of perceived pain throughout image acquisition.

Our finding of bilateral post-surgical rCBF increases in S1 is supported by primate electrophysiological studies of S1 neurones with bilateral receptive fields[51], and other imaging reports of evoked painful and non-painful stimulation of the trigeminal nerve, e.g.[49], [52]. Our observations of bilateral rCBF changes in thalamus most likely relate particularly to representation of pain by the trigeminal system. In particular, crossed and uncrossed somatosensory and nociceptive afferents project from the trigeminal ganglion, via the principal sensory nucleus and nucleus caudalis respectively, terminating at the ventral medial and lateral posterior regions of the thalamus. Both these thalamic regions contain bilateral representations of the intra-oral cavity[53]. In addition, extensive interconnections in thalamus and hypothalamus[8] are likely to underpin bilateral changes in post-surgical thalamic rCBF and may represent changes in arousal as well as the experience of ongoing pain[54].

Demonstration of local increases in CBF in Vp during post-surgical, ongoing trigeminal pain echo recent reports of changes in brain activation in Vp in preclinical studies[55], following hypertonic saline injection to the masseter muscle[56] and following noxious electrical stimulation of the tooth pulp[49]. These findings challenge the traditional belief that Vp is associated only with somatosensation, with nociceptive trigeminal afferents processed only via nucleus caudalis of the trigeminal nerve[53] and provide evidence that Vp plays a role in pain processing. We could not identify rCBF changes in trigeminal nucleus caudalis; this region of hindbrain was inferior to the ASL imaging volume prescribed. Further methodological development is required to include these regions within the imaging volume. We speculate that extended brainstem coverage is likely to improve our ability to detect significant bilateral post-surgical CBF increases in the trigeminal ganglion (TG). While we report a cluster of significant CBF increase in left TG only, CBF increases in right TG were slightly below statistical cluster threshold and are likely to be explained by type-II error. Our findings of CBF changes in response to pain in the mandibular branch of TG are contrary to a recent report using BOLD-fMRI, which reported signal changes in the maxillary branch of TG only[49].

Taken together, our findings have potential to impact positively upon the role of neuroimaging in assessing novel treatments for pain[57]. We conjecture that in future, pCASL-derived rCBF measures might be used as prospective independent endpoints for pain assessment, rather than an adjunct to patient self-reported pain. We acknowledge such a statement is likely to provoke considerable controversy within the field[58]. In common with previous reports, for example [59], our findings of correlations between post-surgical rCBF and VAS estimates of self-reported pain, limited only to brain regions known to underpin the pain experience, demonstrate that our results are physiologically plausible and relate (at least in part) to the pain experience. Caution should be exercised, however, in over-interpretation of VAS pain-estimate relationships with individual ROIs; first, given the multi-dimensional nature of the pain experience [60] multivariate regression analyses are likely to provide better predictions of verbal response[61]; secondly, seeking only to replicate patient-self reported endpoints using neuroimaging obviates its use. Arguably imaging-based markers of ongoing pain should be considered in terms of their ability to add value over and above self-report[57].

Our finding of reproducible rCBF data, within and between sessions, makes ‘cross-over’ assessments of pain treatments tenable. A critical next step to develop ASL as a methodology for assessing modulation of ongoing pain will be to demonstrate pain-related CBF changes that are attenuated by an analgesic of known efficacy. Successful demonstration of analgesic-modulated CBF changes should provide the evidence necessary to refine decision-making techniques for assessing efficacy of novel interventions. We envisage several potential uses for the pCASL methodology[57]; central effects of pain medications unrelated to their analgesic action could be assessed in pain-free participants[62]; putative mechanisms of action for novel analgesics might be investigated and possible new indications for existing compounds in related therapeutic areas uncovered; examinations of differential efficacy across pharmacological classes and doses could be realistic applications. In addition, availability of ASL in preclinical MRI should facilitate translational research; ASL studies might potentially illustrate new insights in ongoing pain in preclinical cohorts in which examination of simple behavioural endpoints in response to evoked pain has predominated to date [63].

Improved knowledge of acute ongoing pain should impact upon understanding the central representation of chronic pain; bridging this gap might facilitate developing new medications for intractable pain conditions that are often resistant to currently approved analgesics[64]. Given increasing evidence for changes in brain function and structure relating to chronicity of pain[12], a better understanding of disease-specific ‘neurosignatures’ will be imperative. The ROI-based methodology described here is appropriate to examining post-surgical pain in healthy volunteers, but cannot be applied universally to all persistent pain states; instead, selecting a set of *a priori* ROIs based on previous knowledge of the specific pain condition should be preferred. While we believe ASL has utility in analgesic trials, the method should be equally applicable to assessing changes in ongoing pain in other, non-pharmaceutical scenarios; for example, pain modulation following cognitive behavioural therapy[65]. Additional applications might include assessing pain in individuals less able to verbalise self-reported pain, for example children[66] or potentially, patients with consciousness disorders[67].

In summary, using perfusion MRI, in concert with the TME model, we have described a network of rCBF increases representing ongoing post-surgical pain. Post-surgical CBF changes are reproducible within- and between sessions. Our findings represent the beginning of a novel approach to measure ongoing pain as an alternative to self-report. The approach is stable and provides robust, repeatable results in a relatively small group of participants, compared to conventional studies solely using self-reported pain as endpoints[68]. Reduction in study numbers is likely to provide benefits in the early phase assessment of putative analgesics and other interventions, both in terms of cost and time. While we have focussed upon assessment of acute, ongoing post-surgical pain, we believe that developing the methodology for examining pain in patients with persistent painful conditions will be valuable for pioneering much-needed new therapies.
